# Natural Alternatives in the Treatment of Colorectal Cancer: A Mechanisms Perspective

**DOI:** 10.3390/biom15030326

**Published:** 2025-02-24

**Authors:** Karen Vanessa Fernandez-Muñoz, Cuauhtémoc Ángel Sánchez-Barrera, Marco Meraz-Ríos, Jose Luis Reyes, Eloy Andrés Pérez-Yépez, Maria Teresa Ortiz-Melo, Luis I. Terrazas, Monica Graciela Mendoza-Rodriguez

**Affiliations:** 1 Unidad de Biomedicina, Facultad de Estudios Superiores Iztacala, Universidad Nacional Autónoma de México, Avenida de los Barrios 1, Los Reyes Iztacala, Tlalnepantla 54090, Mexico; karen.fernandez@cinvestav.mx (K.V.F.-M.); angel.casb94@gmail.com (C.Á.S.-B.); teresaortiz@iztacala.unam.mx (M.T.O.-M.); 2 Departamento de Biomedicina Molecular, Centro de Investigación y de Estudios Avanzados del Instituto Politécnico Nacional, Avenida Instituto Politécnico Nacional 2508, Ciudad de México 07360, Mexico; 3 Laboratorio de Genómica, Instituto Nacional de Cancerología, Tlalpan 14080, Mexico; 4 Laboratorio Nacional en Salud, Facultad de Estudios Superiores Iztacala, Universidad Nacional Autónoma de México, Avenida de los Barrios 1, Los Reyes Iztacala, Tlalnepantla 54090, Mexico

**Keywords:** colorectal cancer, chemoresistance, natural compounds, bioactive compounds, adjuvants, chemosensitivity

## Abstract

Colorectal cancer (CRC) is one of the deadliest neoplasia. Intrinsic or acquired resistance is the main cause of failure of therapy regimens that leads to relapse and death in CRC patients. The widely used chemotherapeutic agent 5-fluorouracil (5-FU) remains the mainstay for therapeutic combinations. Unfortunately, chemotherapeutic resistance and side effects are frequent events that compromise the success of these therapies; the dysregulation of enzymes that regulate 5-FU metabolism increases the expression and activity of efflux pumps. Additional tumor cell adaptations such as epithelial–mesenchymal transition (EMT), autophagy shaping of the tumor microenvironment, and inflammation contribute to chemoresistance. Finding new strategies and alternatives to enhance conventional chemotherapies has become necessary. Recently, the study of natural compounds has been gaining strength as an alternative to chemotherapeutics in different cancers. Curcumin, trimethylglycine, resveratrol, artemisinin, and some helminth-derived molecules, among others, are some natural compounds studied in the context of CRC. This review discusses the main benefits, mechanisms, advances, and dark side of conventional chemotherapeutics currently evaluated in CRC treatment. We also analyzed the landscape of alternative non-conventional compounds and their underlying mechanisms of action, which could, in the short term, provide fundamental knowledge to harness their anti-tumor effects and allow them to be used as alternative adjuvant therapies.

## 1. Introduction

Colorectal cancer (CRC) is one of the main concerns worldwide due to its increasing incidence and mortality rate in the last few years. Currently, CRC is the third most common cancer (1.9 million new cases) and the second leading cause of cancer-related death (930,000 deaths) [1]. Furthermore, the World Health Organization (WHO) estimates that by 2030 there will be 2.2 million new cases of CRC and 1.09 million deaths caused by CRC [2].

The etiological origin of CRC can be classified into two principal groups: hereditary, around 20%, and sporadic, representing 80% of total cases [3]. Sporadic CRC emerges from the accumulation of acquired somatic mutations and epigenetic alterations related to several modifiable lifestyle risk factors, such as obesity, sedentary behavior, high alcohol and tobacco intake, low fiber consumption, high-fat diets, and processed meats [4]. Three main pathways have been described for the formation of sporadic CRC tumors: (I) the adenoma–carcinoma sequence, influenced by chromosomal instability (CIN), microsatellite instability (MSI), KRAS, APC, and Tp53 mutations; (II) the serrated pathway, governed by the methylation of CpG island methylator phenotype (CIMP), MSI, BRAF, and CDKN2A mutations; and (III) the inflammatory pathway, related to the activation of inflammatory signaling pathways such as NFκB, IL-6/STAT3, COX-2/PGE2 and IL-23/Th17 [5,6].

The heterogeneity of tumor origins influences the treatment and prognosis of CRC. The classification and identification of tumor subtypes can improve disease management and predictions [7]. The WHO classifies CRC tumors according to their histological characteristics, including (I) adenocarcinoma, which accounts for about 85%; (II) mucinous carcinoma, which represents around 5–20% of the total cases; (III) medullary carcinoma (4%); and (IV) Signet-ring carcinoma, with less than 2% [8].

The staging-based classification of CRC tumors is supported by the tumor–node–metastasis (TNM) system, where T represents tumor invasion into the layers of the intestinal wall subdivided into T1: submucosa; T2: muscularis propria; T3: mesocolon or mesorectal fat; and T4: perforation of serosa or invasion into other organs. The N refers to the number of lymph nodes with metastases, where N0: no lymph nodes; N1: 1–3; N2: 4 or more. The M indicates the presence of distant metastasis, including M0, which has no distant metastasis, and M1, with metastasis beyond regional lymph nodes. In this context, colorectal tumors are classified as early stage or stage I, where T1 or T2 indicate tumors without metastases, stage II is T3 or T4 tumors without metastases, stage III is tumors with lymph node metastases, and stage IV is indicative of tumors with distant metastases [9]. Since detecting CRC in the pre-cancerous and early stages reduces mortality, periodic screening is recommended for adults aged 50–70. However, due to the increasing incidence of CRC in young patients, recent guidelines recommend screening at age 45 [10]. In this context, the treatment choice and its success depend on the tumor stage at diagnosis.

**Figure 1:**
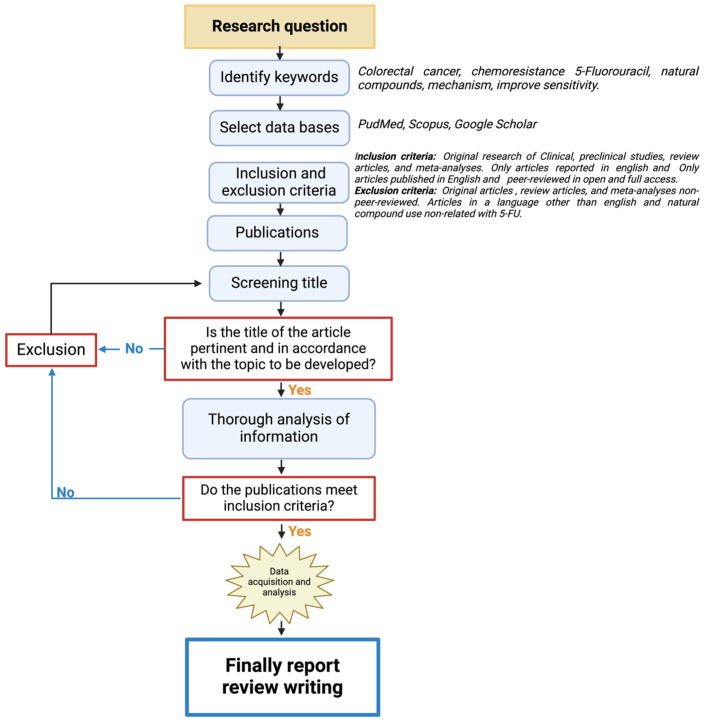
A flowchart of the methodological approach that was followed for the selection/exclusion of the articles.

This is a narrative review structured as shown in Figure 1. First, the keywords related to the research question were identified, and recognized databases were utilized for the search (PubMed, Scopus, and Google Scholar). The inclusion criteria focused on all the works on colorectal cancer treatment and natural compounds and excluded the articles that did not have experimental validation and relation to colorectal cancer treatment with 5-Fluorouracil (5-FU). All the publications selected were thoroughly analyzed in their full-text forms to ensure alignment with the inclusion criteria. This system allowed for the development of the following topics of the review.

## 2. CRC Treatment

The treatment management of patients with CRC mostly depends on the stage of their diagnosis. Surgery is the primary treatment used in the early stages (I and II), and in the advanced stages, surgery accompanied by systemic therapy is the option for patients with positive lymph nodes and patients with a low-burden spread of tumors [11]. Unfortunately, it is well known that more than 50% of CRC patients will develop metastasis, mainly to the liver; in this situation, most tumors are unresectable, and systemic therapy becomes the primary treatment for metastatic CRC (mCRC) [12,13]. Systemic therapy consists of chemotherapy, targeted therapy, and immunotherapy. Chemotherapy is the primary treatment for mCRC inducing cytotoxicity against cancer cells; targeted therapy recognizes specific molecules that are involved in signaling pathways related to proliferation and survival, such as epidermal growth factor receptor (EGFR) and vascular endothelial growth factor (VEGF); and immunotherapy blocks immune checkpoint molecules such as PD-1, PD-L1, and CTLA-4 which restore the activation of adaptative immune cells [14].

5-Fluorouracil (5-FU) is the central axis for therapeutic combinations in advanced CRC treatment. However, individual therapy’s function and success rates are limited (10–15%). Therefore, it is frequently administered together with other drugs, such as leucovorin, oxaliplatin, or irinotecan, increasing the response rates by 40–50% [15]. Nonetheless, it has been determined that 40–50% of all patients with CRC will develop metastasis either at the time of diagnosis or as a recurrent disease after therapy [13,16]. mCRC remains incurable, and the five-year relative survival rate is only about 14% [14].

It is well known that current pharmacological chemotherapeutic drugs are closely related to severe side effects in cancer patients [17]. The adverse effects of chemotherapy in CRC patients include typical symptoms such as asthenia, nausea, vomiting, hair loss, diarrhea, and immunosuppression [18]. However, other symptoms with more significant repercussions on the patient’s health may occur. For instance, the pharmacological scheme of 5-FU plus irinotecan causes intestinal mucositis in 5–15% of patients [19]. Cardiotoxicity and thrombosis have been reported in 5-FU treatment, and oxaliplatin administration can provoke peripheral neuropathy in about 89% of patients [20,21]. Usually, these side effects are intolerable for patients who are forced to reduce chemotherapeutic drug doses, therefore limiting the therapeutic response, or even requiring that they drop the treatment scheme [22], favoring the acquisition of chemoresistance, which is one of the main problems clinicians face in treating and managing CRC patients.

## 3. Chemoresistance in CRC

Chemoresistance is associated with a lack of response to therapy and relapse in CRC patients, representing a significant problem in medical oncology. Drug resistance occurs in about 90% of patients with mCRC, and high mortality rates can be attributed to a deficient response to chemotherapeutics [23,24].

**Figure 2:**
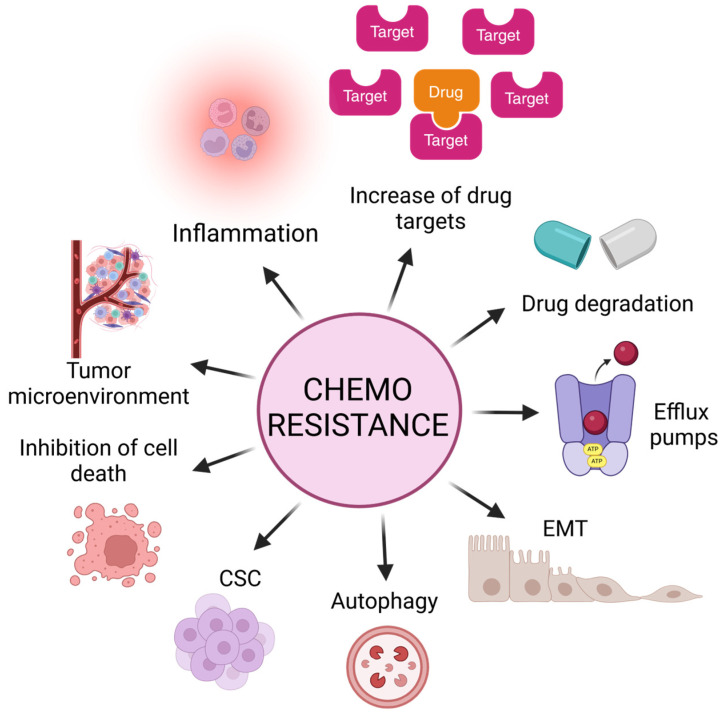
Main mechanisms and alterations in tumors that lead to evasion of cell death and, consequently, chemoresistance and tumor relapse. Inflammation, increment of drug targets, alteration in functions of efflux pumps, acquisition of epithelial–mesenchymal transition (EMT) markers, autophagy, presence of cancer stem cells (CSCs), increment of anti-apoptotic proteins, and tumor microenvironment.

Chemoresistance can be classified into two main categories: intrinsic or acquired. Intrinsic chemoresistance exists before drug treatment, implicating pre-existing genetic and cellular alterations. These mechanisms are critical for the result of the initial response to therapies and may influence subsequent outcomes that lead to acquired resistance [25]. Thus, acquired chemoresistance is the adaptation of cancer cells after treatment exposure, in which these cells display the ability to self-renew and re-initiate the cancer-like parental tumor as well as the expression of a distinctive set of surface biomarkers; this hypothesis proposes the existence of cancer stem cells [25]. Both mechanisms lead to central hallmarks such as the evasion of tumoral cell death by increasing anti-apoptotic proteins, inflammation, increasing potential drug targets, alteration in the functions of efflux pumps, the acquisition of EMT, autophagy, the presence of cancer stem cells (CSCs), and the tumor microenvironment as the primary weapons of tumoral cells in resistance (Figure 2).

### 3.1. Enzymes Involved in the 5-FU Metabolism Processing

**Figure 3:**
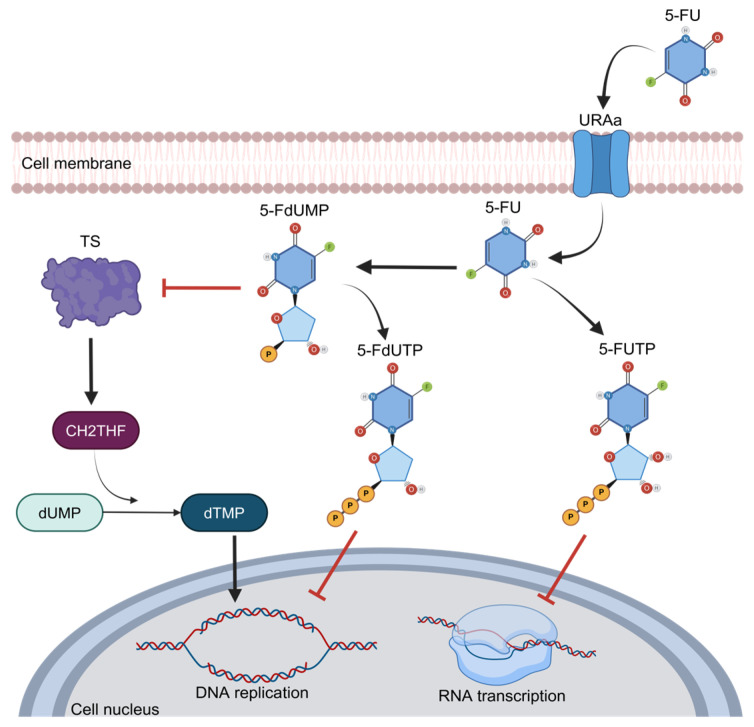
The 5-FU metabolism and inhibition of thymidylate synthase activity.

One of the main features of 5-FU is its functioning as an analog of uracil with a fluorine atom bound at the C-5 position in place of hydrogen that favors cell entry through the exact same facilitated transport mechanism as uracil [26]. Once inside the cells, this compound is converted to 5-fluorodeoxyuridine monophosphate (FdUMP), a potent thymidylate synthase (TS) inhibitor. TS is the main enzyme required for DNA replication, acting as a dimer. It contains a nucleotide-binding site for 5,10-methylenetetrahydrofolate (CH2THF) as the methyl donor necessary for the conversion of deoxyuridine monophosphate (dUMP) to deoxythymidine monophosphate (dTMP), supplying the sole de novo source of thymidylate, which is required for DNA replication and repair [15]. This interaction of 5-FU and TS is performed when FdUMP binds to the nucleotide-binding site of TS, forming a ternary complex with the enzyme and CH2THF, thus blocking the binding site of the normal substrate dUMP and blocking dTMP synthesis and DNA replication [27] (Figure 3). Therefore, the alteration and increase in TS expression or mutation in this protein that disables the binding of FdUMP are the most well-established mechanisms of chemoresistance to 5-FU and imply poor prognosis in cancer patients [28]. As observed in breast and prostate cancer patients, the high expression of TS has been associated with a poor prognosis and survival [29,30]. In primary stomach adenocarcinoma, the expression of TS was associated with poor response and survival in patients who received 5-FU as a therapy [31,32]. In CRC, the main neoplasia treated with 5-FU, the landscape is too similar, and the high expression of TS is frequently observed in refractory patients to 5-FU treatment; in contrast, diminished TS expression correlates with a better response to chemotherapeutic treatments [32]. Indeed, when TS is found in low concentrations, CRC patients have a higher 3-year survival rate [33]. On the other hand, when TS is overexpressed, CRC patients usually do not respond to 5-FU-based schemes, such as the FOLFOX regimen (5-FU, oxaliplatin, and leucovorin) [34].

Dihydropyrimidine dehydrogenase (DPD) is an enzyme involved in 5-FU catabolism. In the intravenous administration of 5-FU, a significant percentage of this drug (~80%) is metabolically degraded in the liver by the DPD enzyme, converting 5-FU to dihydro fluorouracil (DHFU), an inactive metabolite unable to stop DNA replication and synthesis due to its inability to join to TS [35]. Therefore, there is an inverse correlation between DPD expression and 5-FU-based chemotherapeutic response; in addition, DPD overexpression is associated with an unfavorable prognosis in different types of cancers, including CRC. The low expression and activity of DPD have been related to a better response to 5-FU in advanced head and neck cancer, and the same has been observed in colorectal cancer, in which the inhibition of DPD levels reverses 5-FU resistance [36].

5-FU is an analog to the nucleotide uracil; thus, its entry into cells is through facilitated transport, and it is then converted to 5-fluorouridine monophosphate (FdUMP) and may act as an inhibitor of DNA replication through the inhibition of thymidylate synthase (TS) activity, binding to the nucleotide-specific site in TS for the attachment of methylenetetrahydrofolate (CH2THF), the methyl donor necessary for the conversion of deoxyuridine monophosphate (dUMP) to deoxythymidine monophosphate (dTMP). This is how it blocks the binding of the normal substrate dUMP as 5-FdUTP. Owing to this, the sole source of thymidylate is blocked; therefore, DNA replication is inhibited. The analogous structure of 5-FU to the nucleotide uracil allows it to disrupt the transcription of RNA.

### 3.2. Efflux Pumps

The activity of efflux pumps also contributes to chemoresistance, and efflux pumps are mainly members of the ATP-binding cassette (ABC) family. These drug transporters increase chemotherapeutic efflux, reducing its intracellular accumulation in cancer cells [37]. There are three main proteins involved in chemoresistance: P-glycoprotein ABCB1 (MDR1), multidrug resistance protein ABCC1 (MRP1), and breast cancer resistance protein ABCG2 [38]. Recently, compelling evidence has demonstrated that the inhibition of these transporters can improve the efficacy of chemotherapy [39]. In CRC, it has been shown that, as a secondary effect, 5-FU treatment positively regulates the expression of ABCB1, ABCC1, and ABCG2a through the activation of the IRE1α-XBP1 pathway; thus, favoring the chemoresistance and inhibition of this protein decreased the ABC transporter expression and in turn restored the efficacy of 5-FU treatment in colon cancer cells [38,40].

### 3.3. Epithelial–Mesenchymal Transition

One of the diverse hallmarks of malignancy in tumor cells is the acquisition of a phenotype of epithelial–mesenchymal transition (EMT). This phenotype is characterized by the loss of cell–cell adhesion and apical–basal polarity and undergoing cytoskeleton reorganization, contributing to increased migration and invasion capabilities being displayed by tumor cells [41]. These changes allow cancer cells to gain the properties of mesenchymal cells, such as a greater capacity for migration and invasion, favoring metastasis [42]. The relationship between EMT and chemoresistance to different drugs, including 5-FU in CRC and other cancers, has been broadly studied [43]. Previous reports showed that markers associated with EMT, such as Twist, Zeb1, and Zeb2, after 5-FU treatments, were overexpressed in resistant colon cancer cells (HT29) compared to non-resistant cells. Hence, they display characteristic features of loose cell–cell interaction: a spindle shape, intercellular spaces, and scattering [44]. In CRC patients receiving 5-FU, leucovorin, and oxaliplatin (FOLFOX), nuclear ZEB2 expression was a marker of a poor response to chemotherapy, favoring metastasis, early recurrence, and reduced survival [45]. Thus, the pharmacological modulation of molecules re-emerging during the EMT represents a potential target for CRC therapy [46].

### 3.4. Autophagy

Autophagy is a fundamental biological process where lysosomes degrade cytoplasmic materials. It is contained in double-membrane vesicles named autophagosomes. Although it is also a type of cell death, it has been shown that tumor cells exhibit increased autophagy as a metabolic adaptation to the harsh conditions present in their surrounding microenvironment. Therefore, this process can be a double-edged sword in cancer therapy because of its tumor suppressor and pro-oncogenic properties [47]. In CRC, the dual role of autophagy in 5-FU-based therapy resistance is no exception. CRC patients overexpressing the autophagy-related gene *HSPB8* have shorter overall survival and disease-free survival, and higher tumor stages and lymph node invasion [48]. In some in vitro models, autophagy activation contributes to 5-FU resistance [49]. However, many studies have established that autophagy induction leads to cancer cell death [50,51]. These controversial roles require further studies to be clarified since several early and late (i.e., 3-MA and chloroquine) autophagy inhibitors exist. However, the lack of consensus impairs the pharmacological targeting of autophagy as a therapy.

### 3.5. Chemoresistance Induced by Components of the Tumor Microenvironment

The tumor microenvironment is a complex interaction of different cell types of the immune system, such as T cells, B cells, tumor-associated macrophages (TAMs), and myeloid-derived suppressor cells (MDSCs), with stromal cells like cancer-associated fibroblasts (CAFs), endothelial cells, and soluble extracellular components as growth factors, as well as cytokines, chemokines, hormones, extracellular matrix, etc. This tumor microenvironment can influence cancer patients’ therapeutic response and clinical outcomes in several ways [52,53].

In CRC, there is evidence that CAFs can promote chemoresistance to 5-FU and oxaliplatin by different mechanisms, promoting cell stemness and EMT [54,55]. Recent reports indicate an intricate regulation of CAFs and malignancy in colon cancer cells through regulating the axis molecules, STATs, involved in the EMT process and the regulation of the malignant tumor microenvironment [56,57]. CAFs secrete the pro-inflammatory cytokine IL-6 that induces STAT3 activation through its tyrosine phosphorylation, leading to translocation to the nucleus and activating pro-survival and anti-apoptotic genes [58]. Previous studies have demonstrated that the inhibition of STAT3 activity directly impacts the levels of anti-apoptotic molecules and renders colon tumor cells susceptible to 5-FU treatment [59]. Another STAT molecule involved in the pathogenesis of CRC is STAT6. Previous reports have indicated the critical role of the signaling of this molecule in tumor growth in colonic tissue in a pre-clinical model of CRC. Interestingly, the absence of STAT6 (STAT6-KO mice) and the pharmacological inhibition of STAT6 phosphorylation significantly reduced the number and size of colonic tumors while diminishing the expression of markers related to EMT, such as β-catenin nuclear expression, and increasing the response to 5-FU [60,61,62]. The regulation of these molecules is closely related to the tumor microenvironment and susceptibility to chemotherapy in CRC, making them an excellent therapeutic target that needs further study. On the other hand, the effect of TAMs can also induce oxaliplatin and 5-FU resistance by maintaining high levels of Cathepsin B, increasing the levels of efflux pump proteins and suppressing caspase-mediated apoptosis, to name a few of its effects [63,64]. The interplay between the tumor and microenvironment impacts the biological characteristics of tumoral cells frequently related to the malignancy of tumors, in which specific factors and signaling mechanisms are dysregulated.

### 3.6. Inflammation

It is well known that inflammation plays a crucial role in cancer initiation, progression, and malignancy [65]. Chronic intestinal inflammation is, without a doubt, a significant risk factor for CRC development and can also be involved in a patient’s prognosis and response to therapy [66]. Inflammation contributes to the drug resistance of tumoral cells by increasing the expression of efflux pumps and altering the levels of drug-metabolizing enzymes. In addition, inflammation can protect cancer cells from drug-mediated cell death by regulating DNA damage repair, activating oncogenic pathways, and modulating the influence of the tumor microenvironment [67,68]. The high expression of inflammatory molecules is associated with the nuclear factor kappa-light-chain-enhancer of activated B cells (NFκB) that is considered a master regulator of the transcriptional activation of a great variety of inflammatory molecules, such as IL-1β, IL-6, COX2, and STAT3, that are closely related to malignancy and drug resistance [69]. Different studies indicate that high expression levels of NFκB are associated with perineural invasion, lymph node metastasis, and pathologic tumor node metastasis in around 62% of analyzed CRC patients [70]. Therefore, targeting inflammation is a great option to help increase therapy effectiveness. Currently, there is much interest in the use of non-steroidal anti-inflammatory drugs (NSAIDs) as possible adjuvants to conventional therapy with promising results. However, the side effects and toxicity of NSAIDs limit the establishment of their use in the clinic [71,72].

All these mechanisms involved in chemoresistance prevent therapy success. Thus, it is necessary to find new alternatives that help to increase the response to chemotherapy by counteracting chemoresistance and avoiding greater side effects and toxicity.

## 4. Natural Compounds as a Promising Therapeutic Option for CRC

The search for new cancer treatment strategies to improve patients’ therapeutic response has led to a broader vision of non-synthetic compounds. In this regard, natural products are a rich reservoir of bioactive compounds with therapeutic potential [73]. Moreover, around 50% of anti-cancer drugs were obtained directly or indirectly from natural products, such as alkaloids, polysaccharides, polyphenols, diterpenoids, and unsaturated fatty acids possessing various structures, among others [74]. The search for molecules of a natural origin has become relevant. It has been suggested that some of these compounds can sensitize cancer cells to chemotherapy, enhancing the effect of drugs and making them more specific to acting only on cancerous cells. Furthermore, natural compounds tend to be multi-target, so they can interfere with different signaling pathways and thus prevent the appearance of chemoresistance [75]. The origin of natural products can be diverse: microbes, plants, and other living organisms, such as protozoa and helminths, all present anti-cancer properties [73,76].

In vitro and in vivo assays have demonstrated the potential use of natural products in inhibiting cancer cell proliferation, cell cycle arrest, and tumoral cell death [77,78]. This does not mean that natural compounds (NCs) alone are the cure for all types of neoplasia. Previous reports have highlighted that the use of these compounds as a single therapy does not seem to be a viable option to cure a disease as aggressive as cancer; nonetheless, their use as adjuvants to conventional chemotherapy could be a promising therapeutic option to increase anti-cancer therapy responses [79]. Curcumin, resveratrol, artemisinin, trimethylglycine, and helminth-derived molecules are common as new NCs with potential uses as adjuvants to improve 5-FU-based therapy in CRC models. The mechanisms underlying their roles in enhancing the effect of chemotherapeutics and their abilities to revert chemoresistance are areas of intense activity.

### 4.1. Curcumin

Curcumin is an NC derived from the root of the turmeric plant (*Curcuma longa*); it has been used for many centuries as a spice and in traditional medicine [79]. This compound has anti-inflammatory, antioxidant, and anti-cancerogenic properties associated with the regulation of different signaling pathways involved in cell proliferation and survival, which make it a promising therapeutic option for being used as an adjuvant with gemcitabine, docetaxel, oxaliplatin, erlotinib, 5-FU, and celecoxib [80,81,82].

The mechanism of action of curcumin against cancer cells involves the negative regulation of NF-κB, EGFR, and PI3K/AKT signaling pathways that lead to the diminished activity and expression of proteins involved in anti-apoptotic processes, such as Bcl-2 and Bcl-xL, while inducing Bax, caspase-3, and p53 pro-apoptotic molecules. These effects have been observed in many types of cancer, mainly in breast, prostate, gastric, pancreatic, lung, and colorectal cancer [83,84]. In CRC, curcumin has been combined with 5-FU-based therapies in different models where it has been demonstrated to potentiate the chemotherapy effect and reverse chemoresistance through diverse pathways, including TS regulation in colon cancer cell lines, due to its inhibition of the nuclear translocation of NFκB and, consequently, the decrease in E2F1 transcription factor, a promoter of cell proliferation [85,86]. Table 1 summarizes the discovered mechanisms of the synergistic effects of curcumin with 5-FU-based therapies in different in vitro and in vivo models of CRC, including apoptosis inhibition, the dysregulation of survival pathways associated with inflammation such as NFκB and COX2, and the inhibition of stemness and EMT properties.

**Figure 4:**
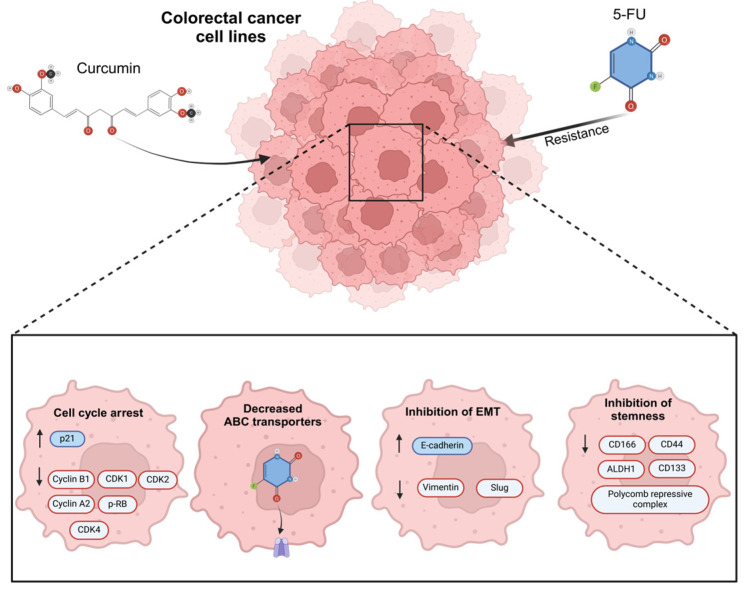
The main mechanisms of action related to the effect of curcumin in 5-Fluorouracil (5-FU) resistance colorectal cancer cells. Previous studies have verified the use of curcumin in 5-FU and its effects as regulator of cell cycle arrest, increasing P21 protein expression and the negative regulation of cyclin B1, cyclin-dependent kinases 1, 2, and 4 (CDK1, CDK2, CDK4), cyclin A2, and the phosphorylation of retinoblastoma, decreasing the expression of ABC transporters, regulating EMT markers such as E-cadherin positively, and reducing Vimentin and Slung proteins. Finally, curcumin has an important effect on regulating stemness markers such as CD116, CD44, ALDH1, CD33, and the polycomb repressive complex.

Several studies in CRC cell lines have shown that induced 5-FU resistance is reverted with exposure to curcumin through cell cycle arrest, decreased ABC-transporter protein levels, the inhibition of stemness, and a reduction in EMT; the mechanisms related to the activity of curcumin in revert 5-FU resistance (Figure 4) [93,94]. In oral cancer, using curcumin as a treatment has been demonstrated to significantly diminish EMT features via c-Met blockade and the inhibition of the ERK effector pathway [106].

Recently, different target drug delivery systems of curcumin and 5-FU have been tested to increase their specificity and cytotoxicity and reduce side effects. Moreno-Quintero et al. (2023) designed 5-FU–curcumin hybrids, tested in the malignant colorectal cancer cells SW-480 and SW-620, and showed that combinatory therapy was more effective in tumoral cells than individual treatment [103]. New alternatives for drug delivery have been proposed to increase the effects of curcumin together with 5-FU, folic acid (FA)-modified nanoparticles, or micelle-crosslinked hydrogel with 5-FU, and curcumin has been tested in HT-29 CRC cells, showing a more significant inhibitory effect than free drugs [102].

The combination of curcumin and 5-FU in animal models demonstrated that this combinatory therapy decreases the tumor volume and weight in CRC xenotransplant models [94,100]. In addition, this combination significantly reduced metastasis to different organs, such as the liver, intestine, spleen, and rectum, in an orthotopic transplant by decreasing angiogenesis and the number of NFκB, COX-2, cyclin D1, c-Myc, ICAM-1, MMP-9, CXCR4, and VEGF molecules [104].

In humans, a randomized Phase II trial evaluated the combination of curcumin with FOLFOX (folinic acid, oxaliplatin, and 5-FU) in a cohort of patients with metastatic CRC, showing a better survival in the FOLFOX plus curcumin treatment in comparison to patients receiving FOLFOX as a single therapy. This combined therapy was safe and well-tolerated, reducing neuropathic pain and neuronal functional abnormalities; however, no conclusive results were obtained regarding effectiveness; hence, more evidence is needed in clinical trials [99].

All these studies indicate that curcumin strongly enhances the effects of 5-FU-based chemotherapy. It avoids chemoresistance mainly by reducing inflammation and tumor cell stemness and enhancing apoptosis cell death, making curcumin a valuable candidate for use as a natural adjuvant in CRC therapy.

### 4.2. Resveratrol

Resveratrol is a polyphenol abundantly present in grape skin and seeds but also in peanuts, berries, tea, and wine [107]. Resveratrol has been reported to have anti-inflammatory and antioxidant activities that confer anti-aging and anti-cancer properties [108]. Its anti-cancer properties have been demonstrated against many types of cancers, such as breast, prostate, liver, and CRC, among others [109,110].

The administration of resveratrol as monotherapy induces apoptosis and autophagy and inhibits tumor growth in in vitro and in vivo models of CRC [111,112]. It is essential to point out that autophagy displays dual roles in cancer treatments, being both related to resistance and an inductor of death in tumoral cells. Resveratrol has demonstrated potential use in inhibiting the progression of colorectal cancer through autophagy-related apoptosis using a SIRT1/FOXQ1/ATG16L pathway axis [112]. In cisplatin-resistant human oral cancer CAR (a tongue squamous cell carcinoma cell line derived from the CAL 27 cell line), the resveratrol treatment induces cell death by an autophagic pathway, which ultimately converges with apoptotic cell death [113]. The dual role of autophagy in response to chemotherapies appears to be dependent on the origin of the neoplasia and used drugs, as observed in p53-null CRC cell lines, in which irinotecan treatment increases the autophagy favoring resistance to tumoral cell death, while data observed in hepatocellular carcinoma, colon, and pancreatic cancer showed that the restoration of autophagy favored 5-FU activity [114,115]. Therefore, the evidence suggests that resveratrol could be an inductor of autophagy and could act synergistically with 5-FU to improve the efficacy of the drug.

Several in vitro and in vivo studies reveal alternative and different mechanisms used by resveratrol to enhance 5-FU activity and cell cycle arrest and increase apoptotic proteins, mitochondrial oxidative stress, and proteins related to the integrity of cell junctions, increasing E-cadherin and claudin-2, among others [116,117,118,119]. The combination of 5-FU plus resveratrol leads to caspase-6 cleavage, increasing cancerous cell death and centrosome amplification, making CRC cells more sensitive to resveratrol and DNA damage [116,117]. Furthermore, the use of resveratrol in induced chemoresistance to the 5-FU cell line (HCT-116R) diminished the hypoxia-inducible factor (HIF) that is associated with poor prognosis in CRC patients [118]. Meanwhile, in a N-methyl nitrosourea-induced CRC model in rats, the administration of 5-FU together with resveratrol ameliorated the histopathological lesions of colon tissues and attenuated inflammation and epithelial damage, increased antioxidant molecules as superoxide dismutase (SOD) and oxidant marker malondialdehyde (MDA), and advanced oxidation protein products (AOPP) in the colon, favoring the anti-tumor response [119]. All these results and the most relevant mechanisms are presented in Table 2.

In summary, the combinatory treatment of resveratrol and 5-FU can potentially enhance the apoptotic effect of 5-FU, regulate molecules involved in oxidative stress and autophagy, and reduce EMT markers, stemness, cell proliferation, and inflammation.

### 4.3. Artemisinin

Artemisinin is a sesquiterpene lactone isolated by the Chinese Professor Tu Youyou from sweet wormwood leaves (*Artemisia annua* L.). Initially, artemisinin and its derivates were used as antimalarial drugs. However, these compounds demonstrated immunosuppressive effects primarily associated with the suppression of T cell proliferation and the downregulation of pro-inflammatory cytokines such as IL-2 and IFN-γ [129,130]. Additionally, these NCs reduced the symptoms of autoimmune diseases in murine models of atopic dermatitis, experimental encephalomyelitis, systemic lupus erythematosus, and ulcerative colitis [131,132]. The immunosuppressive effects of artemisinin and its derivates have been tested in murine models of ulcerative colitis and colitis-associated colorectal cancer. First, in dextran sodium sulfate (DSS)-induced colitis, artemisinin and its derivates decreased the disease activity index by downregulating pro-inflammatory cytokines such as IL-12 and TNF-α, recruiting alternatively activated CD206+ macrophages, and blocking the TLR4 signaling pathway and NFκB signaling pathway, which consequently decreased the number of pro-inflammatory cytokines such as IL-1β, IL-6, and TNF-α. In recent reports, artemisinin and its derivates directly affected the human colorectal cancer cell lines HCT-116 and RKO by inducing apoptotic processes [133,134].

Artemisinin may also function as a chemosensitizer, as reported in esophageal cancer EC109 cells, where exposure to artemisinin reduced the activity of the Wnt/β-catenin signaling pathway, one of the main drivers associated with resistance to chemotherapies, favoring the effect of oxaliplatin [134], and, as with resveratrol, because of its additional immunosuppressive and anti-inflammatory effects, artemisinin can be considered as a potential drug repositioning candidate due to its targeting of autophagy to induce cancer cell death [135].

However, there is limited evidence for the role of artemisinin in regulating chemoresistance processes. Still, its potential beneficial activities, such as having fewer side effects than traditional cancer treatments and its beneficial use in early cancer development, need more studies to establish direct participation in these processes and their interaction with conventional therapies.

### 4.4. Helminth-Derived Molecules

Infections with parasites are more prevalent in developing countries than industrialized ones, correlating with the hygiene hypothesis [136]. The relationship between parasites and CRC has been controversial in the last ten years [137]. Clinical observations suggest that patients infected with *Echinococcus granulossus* have a lower probability of colon cancer development [138]. In concordance with this, it has been shown that early treatment using molecules excreted/secreted by *Taenia crassiceps* (TcES) in a colitis-associated colon cancer (CAC) mouse model leads to reduced pro-inflammatory and proliferative signaling pathways related to cancer establishment and progression, such as STAT3/NFκB, indicating that these molecules may be an excellent early adjuvant therapy in experimental CRC treatment [76]. Using other helminth antigens in vitro, such as those from *Heligmosomoides po-lygyrus*, increased the levels of P53 and P21 proteins in CRC cell lines from mice and humans [139]. These remarkable effects of helminth-derived molecules on CRC were reinforced when used as adjuvant therapy with 5-FU in a mouse model of CAC and the human colorectal cancer cell lines HCT-116 and RKO. Mice receiving the combination of TcES plus 5-FU developed a significantly lower colon tumor load than mice receiving single 5-FU. Moreover, this combination improved the recruitment and activity of NK cells and P53 proteins, inducing pro-apoptotic and anti-proliferative effects [140]. Previously, TcES had been demonstrated to inhibit STAT1 phosphorylation in vitro by activating protein tyrosine phosphatases, such as SHP1, in human and mouse cells [141]. Further research must determine whether this mechanism happens when TcES inhibits STAT3 phosphorylation as an adjuvant to 5-FU during CAC.

On the other hand, human CRC lines HCT-116 and RKO exposed simultaneously to TcES and 5-FU displayed lower proliferative rates and significantly reduced their migration activity [139]. Collectively, these studies show evidence of the potential use of some helminth-derived molecules as an alternative adjuvant to enhance 5-FU activity in the therapy of colorectal cancer. These molecules may negatively regulate both STAT3 and NFκB signaling, altering the interaction between tumoral cells and their microenvironment, reducing the acquisition of malignant phenotypes in tumoral cells, favoring response to therapies, and improving survival.

### 4.5. Trimethylglycine

**Figure 5:**
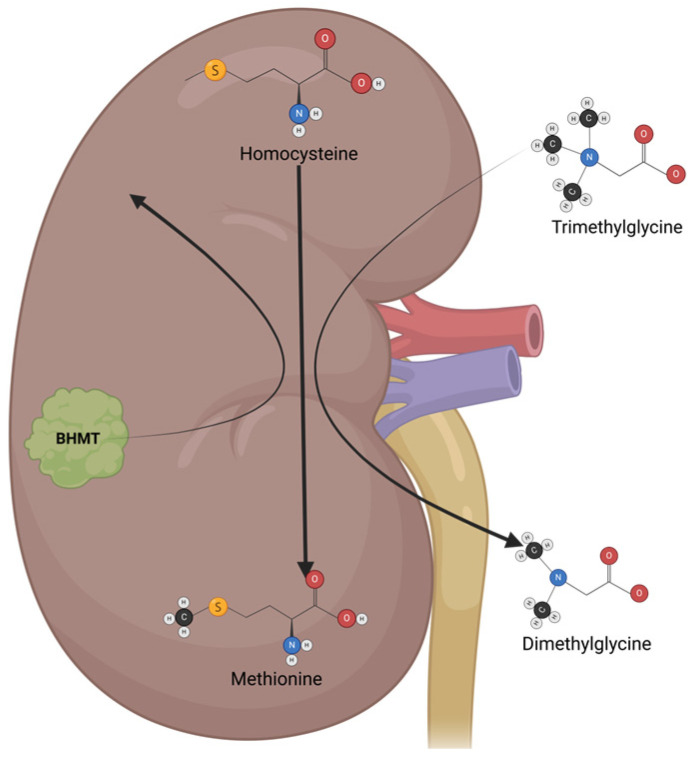
The mechanisms of action and metabolism of TMG. TMG is an osmolyte that functions mainly in the kidney, protecting renal cells from high urea levels and electrolytes. TMG acts as a methyl group donor, switching from trimethylglycine to dimethylglycine, which helps to convert homocysteine to methionine via betaine–homocysteine methyl transferase (BHMT).

Trimethylglycine (TMG), or betaine, is a glycine-methyl derivative with a zwitterionic quaternary ammonium structure. It was first discovered in sugar beets (*Beta vulgaris*). It is also found in high quantities in spinach, wheat germ, wheat bran, and seafood, and is part of the metabolism of microorganisms, plants, and animals. TMG has two main physiological functions: the first is to act as an osmolyte to protect cells from stress conditions, and the second is to act as a methyl group donor [142]. In humans, TMG osmolyte function is mainly executed in the kidneys, which protects renal cells from high levels of urea and electrolytes. TMG acts as a methyl group donor mainly in the liver, where it helps to convert homocysteine to methionine via betaine–homocysteine methyl transferase (BHMT) (Figure 5) [143]. Besides its physiological functions, TMG has antioxidant and anti-inflammatory properties, making it a great option for cancer chemoprevention and cancer treatment in neoplasia with inflammatory components [144].

Some reports have indicated the potential role of TMG as a cancer chemopreventive. A meta-analysis study found a relationship between TMG levels ingested in the diet and the reduced incidence of different types of cancer, especially CRC [144]. An in vivo model of CAC indicated that the administration of TMG in the early stages of the disease significantly reduced the inflammation and development of tumors in the colon [145]. As observed in prostate and lung cancer cell lines, treatment with different doses of TMG reduces oxidative stress and inflammation, favoring the apoptosis process through the negative regulation of NFκB [146,147]. Therefore, it is possible to suggest that TMG, an NFκB activity inhibitor, could regulate mechanisms in resistance to 5-FU, such as TS expression, similarly to curcumin and resveratrol. There is evidence, shown in an in vivo CAC model, that using TMG in combination with 5-FU increases the effectiveness of 5-FU by suppressing the indirect markers of NFκB activity, such as SNAIL1 and β-catenin nuclear translocation, and recovering E-cadherin protein levels; additionally, the use of TMG in this model was shown to significantly reduce the number and size of tumors, as a potential mechanism of the downregulation of STAT6 activity [60]. Thus, TMG is an attractive molecule to be used as an adjuvant for cancer therapy; additionally, previous advantages have been reported in its apoptosis induction of tumoral cells and its anti-inflammatory effect. TMG is considered a safe compound that ameliorates the secondary effects of chemotherapy, such as nephrotoxicity and hepatic damage caused by oxaliplatin treatment [148,149]. Currently, there is limited research using TMG together with conventional chemotherapeutics. Thus, further in vitro and in vivo studies are necessary to translate these results into benefits for colon cancer patients. Nevertheless, there is strong evidence suggesting that TMG could be a great candidate as adjuvant therapy for different types of cancer.

## 5. Conclusions

Colorectal cancer has become a concerning disease in recent years; the high mortality rates of nearly 50% of the diagnosticated patients reflect mainly two problems: (1) a delayed time of diagnosis leading to advanced stages of CRC; (2) the failure of conventional schemes of therapeutic regimens to overcome acquired or intrinsic resistance that contributes to relapses, metastasis, and, finally, mortality in the patients. The current chemotherapeutics—5-FU, oxaliplatin, and folinic acid—are clinicians’ best weapons to fight advanced CRC. Improving these chemotherapeutic treatments and avoiding their most significant chemoresistance problems would benefit patients’ health [150].

Natural compounds have recently attracted attention, given their functions as cancer preventives and potential adjuvants in chemotherapy. NCs have displayed direct and indirect effects in regulating frequent events characteristic of intrinsic and acquired chemoresistance, such as increased drug metabolism, regulated efflux pumps, autophagy, and the acquisition of EMT markers induced by inflammation and the tumor microenvironment [74,75]. Curcumin, resveratrol, artemisinin, TMG, and some molecules derived from ancient parasites like helminths, are natural compounds that have demonstrated their safe use in combinatory therapy together with 5-FU, improving the anti-tumoral response or restoring sensitivity to the drug (Figure 5). All these data support the idea that most NCs, where safe doses for use have been proven, could be postulated as potential compounds for drug repositioning.

**Figure 6:**
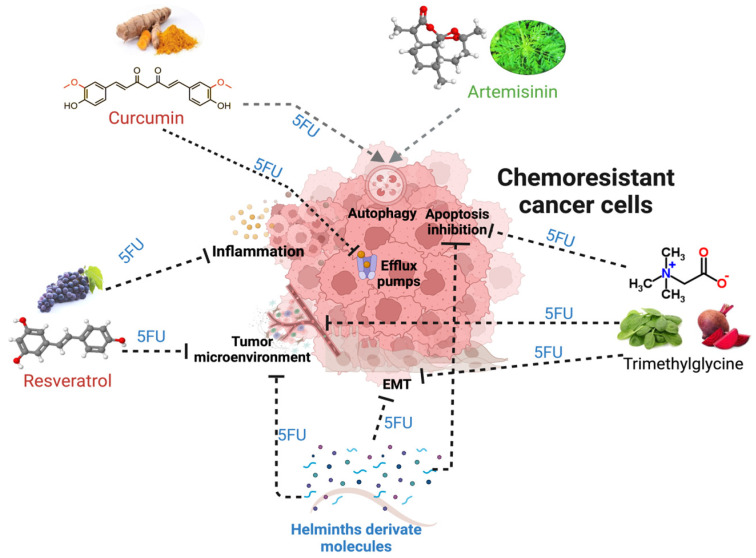
The mechanisms and alterations in tumors that lead to chemoresistance and its potential regulation by natural compounds, such as trimethylglycine, curcumin, resveratrol, artemisinin, and, recently, helminth-derived molecules together with 5FU. The black dotted lines indicate the blocking effect of a natural compound, while the gray lines with arrowheads show the induction of the mechanism related to better responses to treatments.

Interestingly, many of these natural alternatives share similar mechanisms of action, such as STAT signaling modulation and NFκB inhibition, and may have less toxicity than synthetic drugs. These characteristics make NCs an attractive field of research to support their use as adjuvant therapies against colorectal cancer in more preclinical and clinical studies (Figure 6).

## Figures and Tables

**Table 1:** Effects of administration of combined curcumin and 5-FU-based therapies in different CRC models.

Cell Lines			
Author	Model	Condition	Mechanism
[87]	HT-29	20 µM Cur + 50 µM 5-FU	Synergistic effect ↓ COX-2
[88]	HCT-116	10 µM Cur + 200 µM 5-FU + 5 µM OXA	↑ sensitivity to chemotherapeutics and apoptosis ↓ p-EGFR, p-HER-2, p-HER-3, p-IGF-1R, p-AKT, and COX-2
[89]	HCT-116	20 µM Cur + 50 µM 5-FU + 1.12 µM OXA	↓ stemness (↓ CD166, CD44), colony formation, and EGFR. ↑ EGFR promoter methylation
[90]	HCT-116	25 µM Cur + 50 µM 5-FU + 1.25 µM OXA	↓ cell survival, p-EGFR, p-HER-2, p-IGF-1R, p-AKT, COX-2
[91]	HCT-116	5 µM Cur + 1 mM 5-FU	Cell cycle arrest in S-phase ↑ apoptosis ↓ NF-κB, Src, PI3K
[92]	HCT-8/VCR	Different concentrations	Enhanced 5-FU sensitivity
[93]	HCT-116/5-FUR	20 µM Cur + 5 µM 5-FU	↑ sensitivity to 5-FU, apoptosis, and colonosphere formation, and ↓ stemness (↓ CD133, CD44, ALDH1)
[94]	HCT-116/5-FUR and SW480/5-FUR	10 µM Cur + 10 µM 5-FU	↑ sensitivity to 5-FU, apoptosis ↓colony number, stemness (↓ polycomb repressive complex), EMT, and cell cycle arrest.
[95]	Co-culture of HCT-115 and MRC-5	5 µM Cur + different concentrations of 5-FU	↓ colony number, stemness (↓ CD133, ALDH1, CD44), MMP13, NF-κB-p65, p50, TGF-β3, TGF-βR, Smad2, and mesenchymal properties (↓ vimentin, Slug, ↑ E-cad)
[96]	HCT-116/5-FUR in alginate-based 3D culture	5 µM Cur + 0.01 nM 5-FU	↑ sensitivity to 5-FU, apoptosis ↓ cell viability, migration, CXCR4, MMP9, p-NF-κB-p65
[97]	HCT-8/5-FUR	10 µM Cur + 10 mM 5-FU	↑ sensitivity to 5-FU, apoptosis ↓ Nrf2 signaling
[98]	HCT-8/5-FUR	Different concentrations	↑ sensitivity to 5-FU, apoptosis ↓ P-gp and HSP27 G0/G1 cell cycle arrest
[99]	HT-29	30 µM Cur + 20 mg/L 5-FU	Cell cycle arrest in G1/S and G2/M, ↑ p21, ↓cyclinB1, CDK1, cyclin A2, CDK2, p-Rb, cyclin D1, CDK4
[99]	SW480 NNMT overexpression	10 µM Cur + 10 mg/L 5-FU	Cell cycle arrest in G1/S and G2/M ↓ cyclin B1, CDK1, cyclin A2, CDK2, p-Rb, CDK4
[100]	SW620	10 µM Cur + 5 µM 5-FU	↑ apoptosis ↓ cell viability, pERK, pSTAT1, L1
[101]	HT-29	Drug-loaded hydrogels with 60 mg/mL Cur + 5 mg/mL 5-FU	Synergism effect, cell cycle arrest ↑ apoptosis
[102]	HT-29	Nanoparticles GO-Alb-Cur-5FU-FA with 32 μg/mL Cur + 200 μg/mL 5-FU	Potentiates cytotoxicity ↑ apoptosis
[103]	SW480 and SW620	Hybrid 6a, 6d, 6e from 0.625 to 40 µM	↓ cell viability ↑ apoptosis cell cycle arrest
**Animal models**			
Author	Model	Condition	Mechanism
[104]	Orthotopic transplant of HCT-116	60 mg/kg, gavage, twice/week of 5-FU (capecitabine) + 1 g/kg, gavage, daily, of cur, for 28 days	↓ tumor volume; ascites; metastasis to liver, intestine, spleen, and rectum; proliferation index; angiogenesis; NF-κB; COX-2; cyclin D1; c-Myc; ICAM-1; MMP-9; CXCR4; VEGF ↑ apoptosis
[94]	Xenotransplant-HCT-116/5-FUR	20 mg/kg once every 2 days of 5-FU + 50 mg/kg daily of cur, ip. for 40 days	↓tumor volume and tumor weight
[100]	Xenotransplant-SW620	Not specified	↑ cell death and survival rates ↓ tumor volume and proliferation
[105]	Titanium dioxide with dimethylhydrazine-induced CRC	50 mg/kg 5-FU coated with pectin + 200 mg/kg cur coated with pectin	Ameliorate histopathology

Cur: curcumin; 5-FU: 5-Fluorouracil; OXA: oxaliplatin; VCR: vincristine resistant; NNMT: Nicotinamide N-methyltransferase; 5-FUR: 5-Fluorouracil-resistant; GO: graphene oxide; Alb: albumin; FA: folic acid; ip: intraperitoneal.

**Table 2:** Effects of resveratrol and 5-FU administration in combination therapy in different colorectal cancer models.

Cell Lines			
Author	Model	Condition	Mechanism
[116]	HCT-116 p53^+/+^ and HCT-116 p53^−/−^	5-FU IC_50_ + RSV 25–200 µM	↑ cleaved caspase-6 and apoptosis G1 cell cycle arrest
[117]	HCT-116 p53^+/+^ and HCT-116 p53^−/−^	5-FU 50 µM + RSV 200 µM	↑ cleaved caspase-6, apoptosis, centrosome amplification
[120]	HCT-116	5-FU 0.5 µM + RSV 15 µM	↓ cell viability, cell proliferation ↑ apoptosis (↓ Bcl-xL, ↑ Bax, cleaved caspase-9, -3, -8, and PARP), DNA damage, p-JNK, p38 Cell cycle arrest at S-phase
[121]	HT-29 and SW620	5-FU 10 µM + RSV 100 µM	Sensitivity to 5-FU ↑ mitochondrial oxidative stress, ROS, lipid peroxidation ↓ catalase GPx, SOD, p-AKT, p-STAT3
[122]	HCT-116/HCT-116-5-FUR in a 3D-alginate tumor microenvironment	5-FU 0.01, 0.1 and 1 nM + RSV 5 µM	↓ proliferation ↑ sensitivity to 5-FU, colony number, intercellular junctions (↑ E-cadherin, claudin-2), apoptosis, and cleaved caspase-3 ↓EMT (↓ vimentin, Slug), p-NF-κB-p65, p-NF-κB-p50, IκBα
[123]	SW620	5-FU 10 µM + RSV 10 µM	↓ cell viability ↑ H_2_O_2_ production
[124]	HCT-116/HCT-116-5-FUR in a 3D-alginate tumor microenvironment stimulated by TNF-β	5-FU 1 nM + RSV 5 µM	↑ apoptosis ↓ colony number, stemness (↓ ALDH1, CD44, CD133), p-NF-κB-p65, CXCR4, MMP9, EMT (↓ vimentin, Slug, ↑ E-cadherin)
[125]	HCT-116 and DLD1	5-FU 10 µM + RSV 25 µM	Cell cycle arrest in S-phase ↑ apoptosis ↓ EMT (↓ vimentin, Slug), migration, stemness (↓ CD51, CD44), pSTAT3, pAkt, and telomerase activity
[126]	SW480-CD133^+^ and LoVo-CD133^+^	5-FU 15 μM + RSV 80 μM	↑ sensitivity to 5-FU, apoptosis (↑ Bax)
[127]	HCT-116 and HCT-116-5-FUR	5-FU 2 nM + RSV 5 µM	↑5-FU sensitivity by β1-Integrin receptors ↓ cell viability, cell colony formation, migration and invasion, mesenchymal phenotype, apoptosis, HIF-α expression, and inflammation (↓ p-NF-κB) ↓ vascularization (↓ VEGF), stemness (↓ CD44, CD133)
**Animal models**			
Author	Model	Condition	Mechanism
[128]	N-Nitroso-N-methyl urea induced colorectal cancer in rats	i.p. treatment 5-FU 6.25 mg/kg on days 1, 3, and 5, with cycle being repeated every four weeks for 2 months, and orally treated with RSV dissolved in DMSO: 100µ/kg daily for 2 months.	More intact surface epithelium ↑ normal colon cells, integrity of mucosal architecture ↓ inflammation, severity of colon injury, epithelial loss, inflammatory cell infiltrate, epithelial hyperplasia, irregular crypts, goblet cell, proliferation, COX-2
[119]	N-methyl nitrosourea-induced colon cancer in rats	Colon cancer rats orally treated with RSV (10 mg/kg/day) and i.p. injected with 5-FU (12.5 mg/kg, on days 1, 3, and 5 with the cycle being repeated every 4 weeks) over 4 months	Ameliorate liver function (↓ AST, ↓ ALT), kidney function (↓ urea, ↓ creatinine), AOPP, MDA ↑ SOD, p53 ↓ NF-κB, COX-2 Ameliorate histopathology

5-FU: 5-Fluorouracil, RSV: resveratrol, 5-FUR: 5-Fluorouracil-resistant.

## Data Availability

Not applicable.

## Abbreviations

The following abbreviations are used in this manuscript:

3-MA	3-methyladenine
5-FU	5-fluorouracil
5-FUR	5-fluorouracil resistance
ABC	ATP-binding cassette
ABCB1	ATP-binding cassette subfamily B member 1
ABCC1	ATP-binding cassette subfamily C member 1
ABCG2	ATP-binding cassette subfamily G member 2
Alb	Albumin
ALDH1	Aldehyde dehydrogenase 1
ALT	Alanine aminotransferase
AOM	Azoxymethane
AOPP	Advanced oxidation protein products
APC	Adenomatous polyposis coli
AST	Aspartate transferase
ATG16L	Autophagy related 16-like
Bcl-2	B-cell lymphoma 2
Bcl-xL	B-cell lymphoma-extra large
BHMT	Betaine-homocysteine methyl transferase
BRAF	B-Raf proto-oncogene
CAC	Colitis-associated colon cancer
CAFs	Cancer-associated fibroblasts
CDK1/2/4	Cyclin-dependent kinase 1/2/4
CDKN2A	Cyclin-dependent kinase inhibitor 2A
CH2THF	5,10-methylenetetrahydrofolate
CIMP	CpG island methylator phenotype
CIN	Chromosomal instability
COX-2	Cyclooxygenase 2
CRC	Colorectal cancer
CSC	Cancer stem cells
CTLA-4	Cytotoxic T-lymphocyte-associated protein 4
Cur	Curcumin
CXCR2/4	Chemokine receptor type 2/4
DHFU	Dihydro fluorouracil
DMSO	Dimethyl sulfoxide
DPD	Dihydropyrimidine dehydrogenase
DSS	Dextran sodium sulfate
dTMP	Deoxythymidine monophosphate
dUMP	Deoxyuridine monophosphate
E2F1	E2F transcription factor 1
EGFR	Epidermal growth factor receptor
EMT	Epithelial–mesenchymal transition
ERCC1	Excision repair cross-complementing rodent repair deficiency, complementation group 1
ERK	Extracellular signal-regulated kinase
FA	Folinic acid
FdUMP	5-fluorodeoxyuridine monophosphate
FOLFOX	5-fluorouracil, oxaliplatin, and leucovorin
FOXQ1	Forkhead box Q1
GO	Graphene oxide
GPx	Glutathione peroxidase
HER-2/3	Human epidermal growth factor receptor 2/3
HIF	Hypoxia-inducible factor
HSP27	Heat shock protein 27
HSPB8	Heat shock protein family B member 8
ICAM-1	Intercellular adhesion molecule 1
IFN-γ	Interferon gamma
IGF-1R	Insulin-like growth factor-1 receptor
IL-1β/2/6/10/12/23	Interleukin 1β/2/6/10/12/23
Ip.	Intraperitoneal
IRE1α	Inositol-requiring transmembrane kinase endoribonuclease-1α
IκBα	NFκB inhibitor alpha
JNK	c-Jun N-terminal kinase
KRAS	Kirsten rat sarcoma viral proto-oncogene
mCRC	Metastatic colorectal cancer
MDA	Malondialdehyde
MDR1	Multidrug resistance protein 1
MDR1	Multidrug resistance protein 1
MDSC	Myeloid-derived suppressor cells
MMP9/13	Matrix metalloproteinases 9/13
MRP1	Multidrug resistance-associated protein 1
MSI	Microsatellite instability
NC	Natural compound
NFκB	Nuclear factor kappa-light-chain-enhancer of activated B cells
NK	Natural killer cell
NNMT	Nicotinamide N-methyltransferase
NRF2	Nuclear factor erythroid 2-related factor 2
NSAIDs	Non-steroidal anti-inflammatory drugs
OXA	Oxaliplatin
PARP	Poly (ADP-ribose) polymerase
PD-1	Programmed cell death protein 1
PD-L1	Programmed death-ligand 1
PGE2	Prostaglandin E2
P-gp	P-glycoprotein 1
PI3K	Phosphatidylinositol 3-kinase
pRb	Retinoblastoma protein
ROS	Reactive oxygen species
RSV	Resveratrol
SHP1	Src homology 2 domain-containing protein tyrosine phosphatase 1
SIRT1	Sirtuin 1
SOD	Superoxide dismutase
STAT1/3/6	Signal transducer and activator of transcription 1/3/6
TAMs	Tumor-associated macrophages
TcES	Molecules excreted/secreted by *Taenia crassiceps*
TGF-β	Transforming growth factor beta
TGFβ-R	Transforming growth factor-β receptor
TLR4	Toll-like receptor 4
TMG	Trimethylglycine
TNF-α/β	Tumor necrosis factor alpha/beta
TNM	Tumor–node–metastasis
Tp53	Tumor protein p53
TS	Thymidylate synthase
VCR	Vincristine-resistant
VEGF	Vascular endothelial growth factor
WHO	World Health Organization
XBP1	X-box binding protein 1
Zeb1/2	Zinc finger E-box binding homeobox ½
